# Manually defining regions of interest when quantifying paravertebral muscles fatty infiltration from axial magnetic resonance imaging: a proposed method for the lumbar spine with anatomical cross-reference

**DOI:** 10.1186/s12891-016-1378-z

**Published:** 2017-01-19

**Authors:** Rebecca J. Crawford, Jon Cornwall, Rebecca Abbott, James M. Elliott

**Affiliations:** 1 Institute for Health Sciences, Zurich University of Applied Sciences, Technikumstrasse 81, Postfach, CH-8401 Winterthur, Switzerland; 2 Faculty of Health Professions, Curtin University, Perth, Australia; 3 Graduate School of Nursing, Midwifery and Health, Victoria University of Wellington, Wellington, New Zealand; 4 Department of Physical Therapy and Human Movement Sciences, Feinberg School of Medicine, Northwestern University, Chicago, USA; 5 School of Health and Rehabilitation Sciences, University of Queensland, Brisbane, Australia

**Keywords:** Lumbar spine, Paravertebral muscles, Fat infiltration, Magnetic resonance imaging, Region of interest, Manual segmentation, Multifidus, Erector spinae

## Abstract

**Background:**

There is increasing interest in paravertebral muscle composition as a potential prognostic and diagnostic element in lumbar spine health. As a consequence, it is becoming popular to use magnetic resonance imaging (MRI) to examine muscle volume and fatty infiltration in lumbar paravertebral muscles to assess both age-related change and their clinical relevance in low back pain (LBP). A variety of imaging methods exist for both measuring key variables (fat, muscle) and for defining regions of interest, making pooled comparisons between studies difficult and rendering post-production analysis of MRIs confusing. We therefore propose and define a method as an option for use as a standardized MRI procedure for measuring lumbar paravertebral muscle composition, and to stimulate discussion towards establishing consensus for the analysis of skeletal muscle composition amongst clinician researchers.

**Method:**

In this descriptive methodological study we explain our method by providing an examination of regional lumbar morphology, followed by a detailed description of the proposed technique. Identification of paravertebral muscles and vertebral anatomy includes axial E12 sheet-plastinates from cadaveric material, combined with a series of axial MRIs that encompass sequencing commonly used for investigations of muscle quality (fat-water DIXON, T1-, and T2-weighted) to illustrate regional morphology; these images are shown for L1 and L4 levels to highlight differences in regional morphology. The method for defining regions of interest (ROI) for multifidus (MF), and erector spinae (ES) is then described.

**Results:**

Our method for defining ROIs for lumbar paravertebral muscles on axial MRIs is outlined and discussed in relation to existing literature. The method provides a foundation for standardising the quantification of muscle quality that particularly centres on examining fatty infiltration and composition. We provide recommendations relating to imaging parameters that should additionally inform a priori decisions when planning studies examining lumbar muscle tissues with MRI.

**Conclusions:**

We intend this method to provide a platform towards developing and delivering meaningful comparisons between MRI data on lumbar paravertebral muscle quality.

## Background

Magnetic resonance imaging (MRI) has been used for several decades to examine musculoskeletal morphology and pathology, providing insight into tissue composition, disease characterization, response to injury, and changes due to mechanical stress [1, 2]. Advances in MRI technology have elevated the ubiquitous analysis of skeletal muscle composition, and as a consequence, measures towards quantifying muscle fatty infiltration (MFI) have become widely reported with equivocal results.

While data for age-related, degenerative changes to lumbar bones and joints in asymptomatic people have been published [3], few studies assess age-related alterations in paravertebral muscle morphology [4–7]. Cross-sectional [8–12] and longitudinal studies [13] evaluating paravertebral muscle quality indicate a positive relationship between MFI and low back pain (LBP) (i.e. increased fat infiltration is associated with the presence and severity of LBP). It is also suggested that examining muscle quality through MFI measurement is potentially complimentary, if not more relevant, to measurement of quantity (e.g. cross sectional area and/or volume alone) in the assessment of muscle degeneration [14–17]. However, inconsistent associations are also reported [18] and confounded by normative age-related changes [5, 7, 19], degenerative features of the vertebrae or discs [9, 19, 20], and spinal curvature [21, 22].

While several MRI approaches are possible to measure the water and fat species of skeletal muscle, the contemporary standard for evaluating muscle size and structure is chemical-shift MRI, producing water- and fat-only images from dual- and/or multi-echo acquisitions [11, 23–25]. Excellent accuracy has been shown for manual segmentation based on these imaging techniques against spectroscopy [11] and histology [26], and for some common neuromusculoskeletal conditions [23, 27] including LBP [11, 28]. The chemical shift (DIXON in the Siemens environment, IDEAL [iterative least squares solution] in the General Electric environment, mDIXON (Philips), FatSep™ (Hitachi), or WFS (Toshiba)) method collects data at echo times when fat and water are in-phase and out-of-phase. The data can then be combined to generate a co-registered fat and water image, although this method is not immune to field inhomogeneities. Current methods towards improving the estimation of fat and water images have used the IDEAL method, which has been applied successfully for the liver and musculoskeletal system [29, 30]. Despite such reports and technological advances, the vast majority of population-based studies examining pathoanatomical features of the lumbar spine (e.g. including muscle, other soft-tissues such as the intervertebral disc, and the skeletal vertebral column) use conventional T1-weighted [18, 31] or T2-weighted [13, 32] MRI. While different, and potentially less accurate when compared to chemical-shift imaging [14, 29, 30, 33], the data derived from these investigations represent a resource of immeasurable value to investigators interested in the role of all spinal elements.

Despite the obvious usefulness of assessing spinal muscle quality using MRI, a robust and easily replicated platform for acquiring and assessing imaging data on muscle composition remains elusive. Currently, comparisons between studies are challenging and often not possible when investigators have used different manual, semi-automated, or automated segmentation software, programmes, or methods for defining the regions of interest for each muscle (see Table 1 for a summary of published methods). As such, the aetiological significance of MFI in spinal muscles remains unclear. Moreover, efforts to simulate and model muscle activity in both healthy and clinical populations are dependent on accuracy in defining where tissues with different tensile properties (e.g. muscle versus fatty deposits) are located, and there are no widely adopted or standardized assessment tools currently utilized for this purpose. In order to better understand the influence of MFI content on spinal health, it is imperative that common methodologies are developed and adopted in order to facilitate standardization and accurate comparison of data between studies.

**Table 1:** Representative literature summary of methods in investigations describing paravertebral muscle analysis using magentic resonance imaging (MRI)

Citation	Reliability	MRI Sequence	Slice Selection	Muscles of Interest	ROI Selection	Fat Detection	Measure
Antony et al., 2016 [52]	No	T2 FRFSE	1 slice per level at IVD L3-S1	MF, ES	Semi-Automated interactive Segmentation (intelligent scissors)	Semi-Automated User set pixel intensity threshold	FCSA = (CSAFat)/(CSATotal)
Battaglia et al. 2014 [53]	2 raters (*n* = 25); Qualitative: Intra-rater weighted kappa = 0.71–0.93, Inter-rater kappa = 0.76–0.85; Quantitative: Inter-rater ICC = 0.73–0.90	T1	1 slice per level at IVD L4-5, L5-S1	MF	Qualitative: visual inspection; Quantitative ImageJ manual trace, exclude fascial boder between MF and ES	Qualitative: Goutallier Classification (0,1,2,3,4); Quantitative: pixel intensity range set by user selected fat ROI within MF	% fat = (# of fat pixels)/(total # of pixels)
Beneck et al., 2012 [12]	1 rater; intra-rater: ICC (3,1) = 0.961 for CSA	T1	All slices spanning L4, L5-S1, and S2-S3 vertebral bodies	MF, ES	Slice-o-matic manual trace	Muscle divided into 6–9 regions. Gray-scale signal threshold semi-automatically determined for each region based on user selected small region of muscle	Muscle volume = total volume – fat volume
Bhadresha et al., 2016 [19]	2 raters (*n* = 20); inter-rater ICC = 0.33–0.76, Cronbach’s alpha = 0.59–0.91	T2 FSE	I slice per level at IVD L3-4, L4-5, L5-S1	MF, ES, PS	Visual inspection	18×27 (1 cm apart) grid applied to image. By visual inspection: # of grid points touching fat vs. muscle counted	Muscle to Fat Ratio= Muscle/#fat pixels
Crawford et al., 2016 [5]	No	2-point DIXON (3D fast-field echo T1) whole body	Every 3^rd^ slice L1-L5 (with interpolation to full volume)	MF, ES	Semiautomatic segmentation with linear interpolation (Myrian Intrasense, Paris, France)	Semi-automated ROI from water to fat image	Fat signal fraction (FSF) = (Signalfat/[Signalwater + Signalfat])*100
D’Hooge et al., 2012 [10]	No	T1 FSE	1 slice per level at L3 superior endplate, L4 superior endplate, L4 inferior endplate	MF, ES, PS	ImageJ manual trace	Automated based on pixel intensity gray-scale threshold	MFI = (signal intensity of “lean muscle”)/(signal intensity of user defined fat ROI)
Fortin et al., 2014 [13]	1 rater; intra-rater: ICC = 0.90 –0.96)	T2	1 slice per level at IVD L3-4 and L5-S1	MF, ES, MF + ES	ImageJ manual trace following fascial borders	User selected pixel intensity gray-scale threshold selected from 4–6 sample ROIs within visible ‘lean muscle’	FCSA = (CSAFat)/(CSATotal); Proportion estimate of muscle fat content = Average signal intensity of Total ROI
Hebert et al., 2014 [18]	1 rater; intra-rater (*n* = 30) ICC (3,1) = 0.93, Bias = −0.70, 95% LOA = −8.11–6.72	T1 SE (0.2 Tesla)	1 slice per level at L4 and L5	MF	Manual trace	Custom MatLab script; threshold set from midpoint between histogram peaks for ‘fat and muscle’	% IMAT = CSAFat/CSATotal (IMAT = intramuscular adipose tissue)
Hu et al., 2011 [54]	3 raters (*n* = 29); intra-rater ICC (3,1) for FCSA = 0.832–0.847, for T2 SI = 0.926–0.957; inter-rater CC for FCSA = 0.858–0.894, for T2 SI = 0.891–0.923	T2 FSE	1 slice per level at IVD L3-4, L4-5, L5-S1	MF, ES	PACS workstation manual trace: lean muscle CSA Drawn avoiding visible fat; Fat% - following outer perimeter	PACS embedded ROI and gray-scale histogram software calculated from mean T2 signal intensity	Lean muscle FCSA = Manually traced Muscle CSA Fat Infiltration = T2 signal intensity
Kjaer et al., 2007 [8]	2 raters (*n* = 50); intra-rater k = 0.86; inter-rater k = 0.58	T1 SE (0.2 Tesla)	1 slice per level at IVD L3-4, L4-5, L5-S1	MF	Visual Inspection	Visual categorization: Normal 0–10%, slight 10–50%, severe > 50% fat	Fat infiltration = (0,1,2)
Paalanne et al., 2011 [28]	2 raters (*n* = 35); intra-rater ICC = 0.86–0.88, inter-rater ICC = 0.85–0.87	T1 FSPGR (In-Phase and Opposed-Phase)	1 slice at upper endplate of L4	MF, ES	neaView Radiology; manual trace	Average Signal Intensity	Relative signal loss = (IP SI – OP SI)/(IP SI)*100%
Pezolato et al., 2012 [22]	2 raters (*n* = 10); intra-rater ICC = 0.90–0.94, inter-rater ICC = 0.86–0.83	T2 FSE	2 slices per level at upper and lower endplates of L1-L5	MF, MF + ES	ImageJ manual trace	Grayscale threshold	Fat infiltrate = TotalCSA – FCSA
Ranson et al., 2005 [55]	1 rater (*n* = 6) × 3: average intra-rater ICC = 0.97	T2	1 slice per level at lower vertebral endplate of L1-L5; upper vertebral end plate of L5-S1	MF, ES, PS, QL	ImageJ manual trace following fascial borders	Grey scale pixel intensity range for muscle, fat, and bone were determined from histogram of manual ROIs of “lean paraspinal muscle”, inter-muscular fat, and vertebral body for either: method 1: global grey scale range (0–120); Method 2: slice specific grey scale range	FCSA = TotalCSA of pixels within grey-scale range for fat
Shahidi et al., 2016 [48]	No	T2	1 slice at L4 (to standardize CSA across individuals)	MF, ES	Quantitative manual trace using MatLab	Pixels identified as either fat or muscle by fitting a two term Gaussian model to the pixel intensities histogram of from segmented ROIs; identified intersection where pixel values above classified as fat and pixels below classified as muscle.	Cross Sectional Area Fat signal fraction = *n*pixels fat/*n*pixels fat + *n*pixels muscle
Valentin et al., 2015 [7]	1 rater (*n* = 24); intra-rater ICC = 0.88–0.99	T1	All slices spanning lower endplate of L5 to upper endplate of L1 (10 mm slice thickness)	MF, ES, PS, RA	Analyze Direct Manual trace	Average SI	MFI = (Muscle SI)/(Subcutaneous Fat SI)

*PS* Psoas Major, *MF* Multifidus, *ES* Erector Spinae, *QL* Quadratus Lumborum, *RA* Rectus Abdominus, *FCSA* Functional Cross Sectional Area, *SI* Signal Intensity, *IP* In-Phase (Water), *OP* Opposed-Phase (Fat), *FSPGR* Fast-Spoiled Gradient Echo, *ROI* Region of Interest

We consider that standardized and easily replicated methods enabling consistent MFI quantification are urgently required to facilitate widespread adoption of an agreed technique in measuring muscle quality. While there is a general trend toward optimising automated methodologies that quantify muscle composition based on differential tissue signal intensities, even the newest, time-efficient tools require a degree of manual input in defining regions of interest (ROI) [34–36]. A standardised ROI method is arguably most important for these studies where it has been speculated that difficulties identifying morphology results in poorer repeatability [34]. With continued improvements and uptake of MRI technology, analyses utilizing a common method for the identification of ROI’s could result in increasing insight and clinical translation within this important area of study. The purpose of this proposed method is therefore to provide an option for use as a standardized MRI procedure for measuring lumbar paravertebral muscle composition, and to stimulate discussion towards establishing consensus for the analysis of skeletal muscle composition amongst clinician researchers.

## Method

### Challenges for producing a region of interest of lumbar muscles: Important background for developing the proposed method

**Fig. 1:**
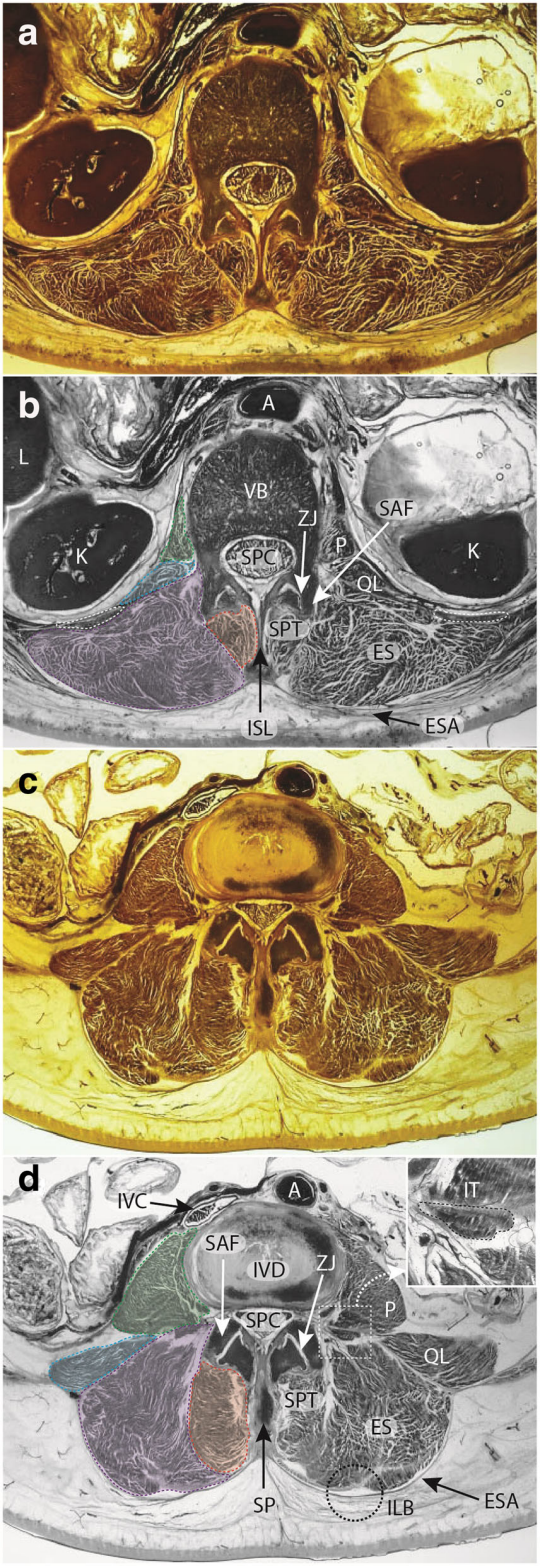
Axial E12 plastinated sections (**a, c**) and schematic illustrations (**b, d**) at approximately L1 (**a, b**) and L4 (**c, d**) highlighting anatomical structures at these vertebral levels. **b, d** Dotted lines and shading, *Green* - psoas major muscle; *Blue* – quadratus lumborum muscle; *Purple* – erector spinae muscles; *Red* – spinotransverse muscles. **b** round white dotted regions (bilateral) denote 12th rib. **d** square dotted box surrounds enlarged inset; round dotted circle indicates morphological feature of interest (ILB fatty ‘tent’). *Legend:* A – aorta; ES – erector spinae muscles; ESA – erector spinae aponeurosis; ILB – iliocostalis – longissimus boundary and indentation; ISL – interspinous ligament; IT – intertransversarii muscle; IVC – inferior vena cava; K – kidney; L – liver; P – psoas major muscle; QL – quadratus lumborum muscle; SAF – superior articular facet; SP – spinous process; SPC – spinal canal; SPT – spinotransverse muscle group; ZJ – zygapophysial joint

Detailed descriptions of the complex anatomy of lumbar paravertebral muscles and definitions regarding the spatial distribution of MFI on axial MRI are limited [37–40]. Published images demonstrating investigators’ definition of ROI for these muscles predominantly depict the lower lumbar levels, with limited identification of separate muscles. Further, descriptions lack details towards acknowledging the complex three-dimensional structure that produces a changing spatial relationship observed across lumbar segmental levels. The lumbar paravertebral muscles typically examined in such studies include: multifidus (MF) as the largest lumbar spinotransverse muscles; erector spinae (ES) including lumbar longissimus and iliocostalis; and less frequently, psoas (including major and minor), and quadratus lumborum (see Fig. 1). This paper intentionally focuses on MF and ES as these are presumed to have the greatest clinical significance. However, other paravertebral muscles exist in the lumbar spine (e.g. the lumbar interspinales and intertransversarii, and thoracic semispinalis), yet they are generally not mentioned in descriptive investigations. This may relate to a lack of image resolution with available sequences, making it challenging to accurately delineate individual muscles from adjacent structures, and it therefore remains unclear how they should be treated when defining ROIs.

Our proposed method outlined in the results section, provides a foundational solution for the problem of how to measure muscles traversing the lumbar spine, and includes suggestions on operational characteristics for acquiring MR images. While we offer this starting point for a common methodology to facilitate accurate definition of lumbar muscle ROI, we are cognisant that the method is not a definitive end-point on ‘how to’. We hope that with time and new research findings these methods will be modified, expanded, and refined.

### Anatomically defining the muscles of interest

Figure 1 presents axial E-12 sheet-plastinates from cadaveric material (A&C) and schematic representations of the same (B&D) for approximately the L1 (A&B) and L4 (C&D) levels to depict the paravertebral muscles and adjacent anatomy for cross reference to the descriptive text to follow. The seminal anatomical studies we use for reference are those of Cornwall et al.[38], Macintosh et al. [39–41], and Bogduk [37].

#### Spinotransverse muscles

This group is located immediately lateral to the spinous processes of vertebrae throughout the length of the vertebral column (Fig. 1b and d). These muscles maintain a consistent morphology in all vertebral regions [38], where fibres in the cervical and thoracic spines originate from the spinous process of the cranial vertebra, to insert into the transverse processes of several more caudal vertebrae. In the lumbar spine, where the transverse processes have evolved to become the mammillary processes (and the costal elements - the ribs - are now the transverse processes), these muscles insert into the mammillary processes of the vertebrae caudal [42]. In the cervical and thoracic spine the longest, multisegment-spanning muscles of this group are named the semispinalis; in the lumbar spine, this entire muscle group is all termed the multifidus (MF) despite the commonality in morphology with spinotransverse muscles in the other spinal regions. The lumbar multifidus occupy the space between the mammillary processes (laterally) and spinous processes (medially), with multiple fascicles originating from each lumbar level. These fascicles pass caudally to insert into vertebrae sequentially, and due to their architecture (fascicles from each segmental level inserting into those that are adjacent via a myomyonal junction) it is extremely difficult to determine where each fascicle has originated when assessing MR images. This is because some individual epaxial muscles (broadly, those muscles which developmentally arise to form the post and paravertebral muscles [43], such as the erector spinae and spinotransverse muscles) are not encapsulated by their own, independent layer of epimysium - a factor which normally assists in identifying or delineating a skeletal muscle as an individual entity [38, 42]. The epaxial muscles are therefore different to hypaxial muscles (those muscles that developmentally include all trunk muscles that are not epaxial in origin) in regards to being able to identify each individual muscle via easily distinguishable borders, and this adds to the problems interpreting MRI of these muscles when a clear fascial boundary does not exist (e.g. different segmental levels of multifidus, or between iliocostalis and longissimus). This means interpretation of muscle quality can be problematic when looking to identify where each multifidus fascicle may be originating or inserting. Other muscles within this space include the interspinales and intertransversarii (see Fig. 1c and d insert), although these are not considered part of the spinotransverse muscle group. The rotatores muscles, apparent in other spinal regions, do not exist in the lumbar spine [38].

#### Lumbar erector spinae

The lumbar erector spinae (ES) is part of a large group that extends the full length of the spine comprising longissimus and iliocostalis and occupying a lateral position compared to the spinotransverse group of muscles (see Fig. 1b and d). In anatomical terminology, the lumbar region is the location of the erector spinae muscles iliocostalis lumborum pars lumborum, and longissimus thoracis pars lumborum. It is difficult to accurately delineate between each of these two muscles on most forms of imaging due to the fact these muscles are not encapsulated with their own sheath of epimysium at their boundary, making visualisation of their anatomical border difficult to distinguish (similar to the problems identifying different segmental levels of multifidus).

## Results

### Defining the regions of interest from MRI

**Fig. 2:**
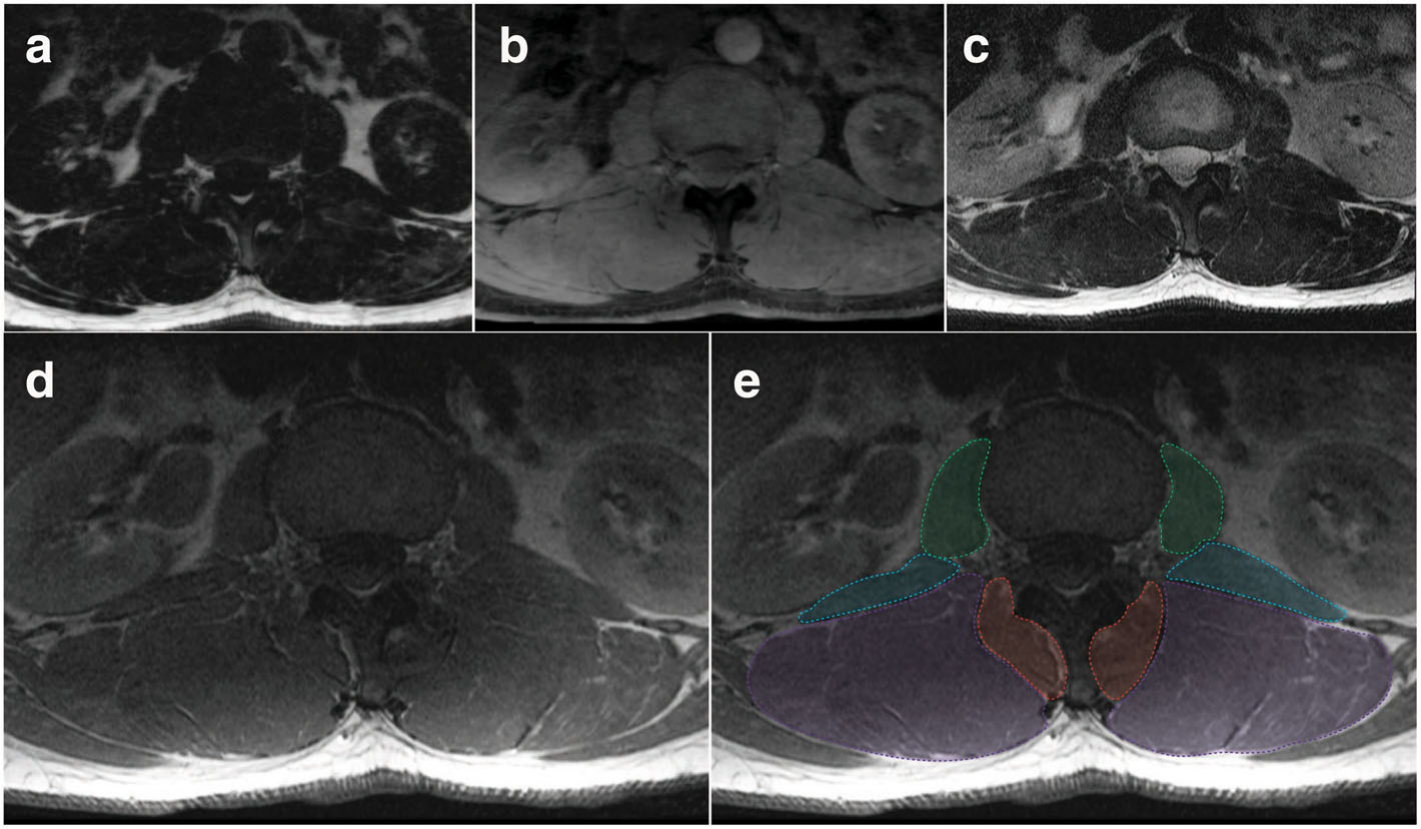
Axial MRIs at the L1/2 disc level of a 47 year old male depicting fat- (**a**) and water-separated (**b**) chemical shift, and T2- (**c**) and T1-weighted (**d** and **e**, same) images. **e** Dotted lines and shading depicting the regions of interest for: *Green* - psoas muscle; *Blue* – quadratus lumborum muscle; *Purple* – erector spinae group (longissimus and iliocostalis together); and *Red* – spinotransverse muscles (predominantly multifidus)

First, in discerning lumbar level from MRI, we suggest using the iliac crest tangent sign [44] by connecting a tangent between the iliac crests, which bisect either the L4 vertebra or the L4/5 intervertebral disc. This should be achieved using the coronal image with cross-reference to the sagittal and then axial images in the full imaging dataset. It should be noted that in following the method outlined, the user may need to scroll between adjacent MRI slices to visualise landmark structures to those on the level being segmented; the method is applicable to studies examining paravertebral ROIs for single or multiple slices. Second, in terms of commencement order for defining separate regions of interest, we recommend a randomised approach for the left or right side, and/or separate muscles as recent evidence has shown their influence on repeatability [34, 45]. Third, definitions for ROI for MF, and longissimus and iliocostalis lumborum (together as ES) are included, describing the medial, anterior, lateral, and posterior borders in order (cross reference to Figs. 2  (L1﻿/2) ﻿and 3 (L4/5)). All muscles are present at the first lumbar level with their distal attachments described.

**Fig. 3:**
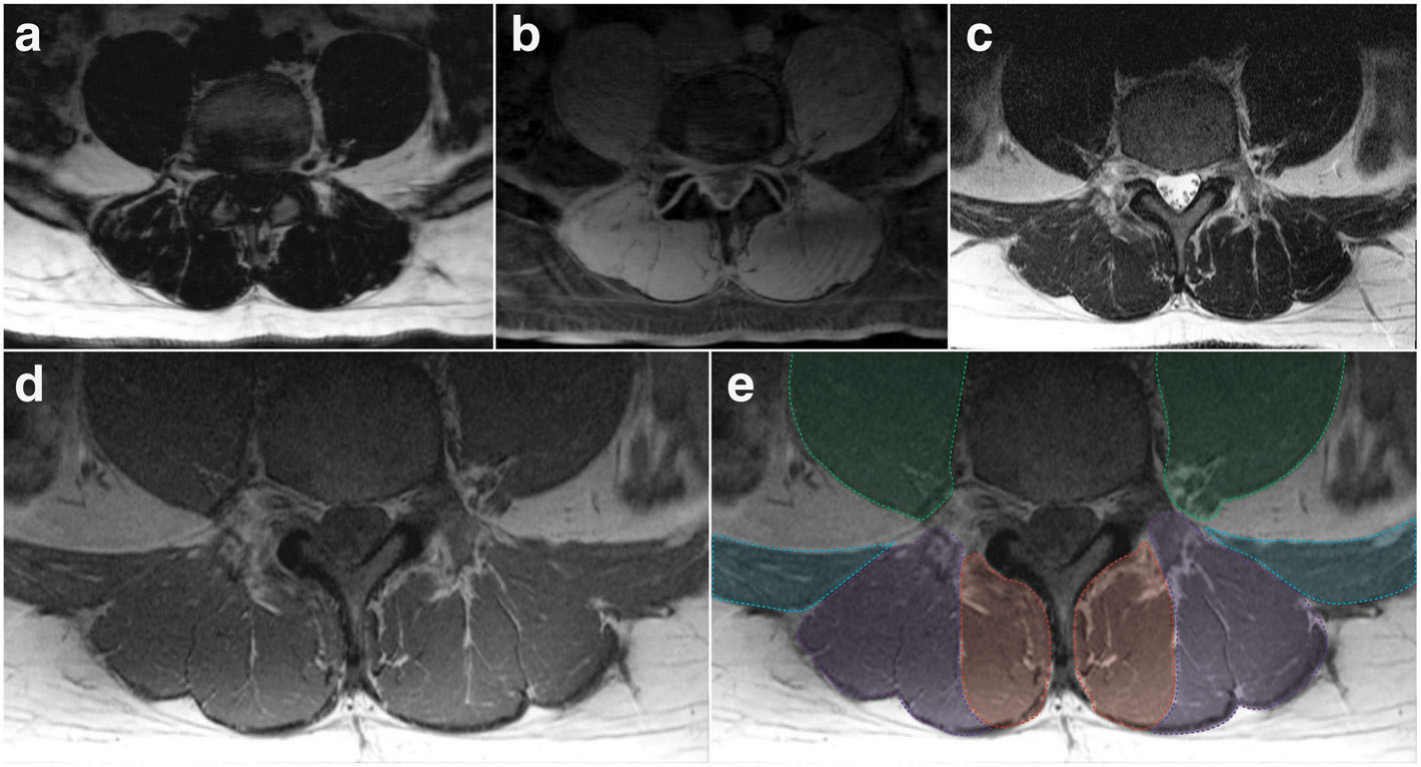
Axial MRIs at the L4/5 disc level of a 47 year old male depicting fat- (**a**) and water-separated (**b**) chemical shift, and T2- (**c**) and T1-weighted (**d** and **e**; same image cropped anteriorly therein truncating psoas) images. **e** Dotted lines and shading depicting the regions of interest for: *Green* - psoas muscle (truncated at the ventral border due to limited visibility in image); *Blue* – quadratus lumborum muscle; *Purple* – erector spinae group (longissimus and iliocostalis together); and *Red* – spinotransverse muscles (predominantly multifidus)

1. Multifidus (MF): medial border is defined by the most superficial aspect of the spinous process following the spinous process deep to where it forms the lamina; the deeper, more anterior border follows the lamina laterally to the anterior aspect of the mammillary process and zygapophyseal joint; the lateral border follows the fascial line (the epimysium of the spinotransverse group) extending from the lateral aspect of the mammillary process between MF and ES toward a small visible indentation at the subcutaneous tissue superficially; the posterior, more superficial border extends along the epimysium of MF that is clearly distinct from the thoracolumbar fascia and adjacent subcutaneous adipose tissue (Figs. 1, 2, and 3). MF is present medially at each lumbar level with increasing volume toward the lower levels of the lumbar spine, with it then diminishing to terminate at its distal attachments to the sacrum where it blends with the lumbosacral fascia. Further technical considerations are included below:

  - This definition is best applied at the lower lumbar levels where distinction between MF and ES is generally clearer (Fig. 1c and d); in the upper lumbar levels (only), thoracic semi-spinalis (ES) will be captured as part of the spinotransverse group due to its medial position. With current technology it is generally not possible to consistently or accurately delineate between semispinalis and multifidus in axial scans through the lumbar spine.
  - Fat approximating the spinous process or lamina is included within the ROI defining MF. For slices transecting the interspinous space, this fat generally overlies the spinous process but remains defined and should be included.
  - When the interspinales muscle and/or interspinous ligament are clearly distinct with a slightly irregular and darkened edge, their lateral contour can be followed rather than the spinous process in defining the medial border.
  - Should deposits of fat be visible at the mid-sagittal line and within the interspinales muscle fibres (rather than in MF), this should not be considered to be MF and therefore excluded from the ROI.
2. Erector spinae (ES): the medial border is defined by the fascial line between MF and ES and abutting the outer aspect of the mammillary process and/or zygapophyseal joint; the anterior border runs along the transverse process laterally as distinct from the quadratus lumborum muscles that lay anterolateral to it and the transverse process, following the anterior thoracolumbar; the lateral border follows the rounded contour of the fascial boundary surrounding iliocostalis; the posterior border follows the ES muscle and aponeurosis as distinct from the thoracolumbar fascia and adjacent subcutaneous adipose tissue; the lumbar intermuscular aponeurosis is included within this ROI (Figs. 1, 2, and 3). The ES longissimus and iliocostalis are present at all levels of the lumbar spine with diminishing size toward the low lumbar levels where they terminate at the posteromedial aspect of the iliac crest and toward the posterior superior iliac spine. Further technical considerations are included below:
  - If distinct, the lumbar intertransversarii that attach adjacent transverse processes should be included as part of the ES (see Fig. 1d square insert).
  - When fat is accumulated between the MF and ES (longissimus), a vertical line from the mammillary process/zygapophyseal joint where a thin fascial line is often present to follow can be used as a guide. When uncertainty exists, take the lateral border of MF.
  - When a large fat-filled ‘tent’ exists between longissimus and iliocostalis at the lumbar intermuscular aponeurosis posteriorly (applicable when including these together as ES), the border is defined by following the most posterior aspect of visible muscle tissue. Refer to Fig. 1d dotted circular line depicting fatty ‘tent’.
  - Particularly at the low lumbar levels, the most anterior fibres of the ES (in the region of the intertransversarii) can be seen to blend with fat tissue in a striated pattern; however, a clear fascial line can direct the ROI.

### MR imaging - operational parameters

There are many different variables that can influence the type and quality of image that is acquired from MR scans, and operational parameters are an important consideration in regards to determining final image output. This method for defining ROI in the lumbar paravertebral muscles has been proposed to facilitate improved comparison between studies, and we consider it important to suggest reporting a minimum set of MRI sequencing parameters. This aspect has been variably described in the literature (refer to Table 1) but in order to improve the study’s relevance to a wider readership, we suggest the following reported inclusions as minimum: Field strength (e.g. 3 Tesla); sequence type (e.g. 2-point DIXON (3D fast-field echo T1) whole body); repetition time (e.g. TR 4.2 ms); echo time (e.g. TE 1.2 and 3.1 ms); flip angle (e.g. 5°); field of view (e.g. FOV 560 × 352 mm); acquired voxel dimensions (e.g. 2.0 × 2.0 × 4.0 mm); reconstructed voxel dimensions (e.g. 1.0 × 1.0 × 2.0 mm); bandwidth (e.g. 120 Hz/Px), acquisition time (e.g. TA 5 min 22 s) and slice thickness (e.g. 4.0 mm).

## Discussion

This paper has proposed a foundational directive (method) for defining lumbar paravertebral ROI’s for studies quantifying MFI from MRI images in the axial plane. We present this method with the aim of standardizing and homogenizing the definition of ROIs for research teams, utilizing several different visual representations of vertebral morphology including E-12 sheet-plastinated anatomical sections, schematic representations of spinal structures, fat- and water-based chemical-shift MRIs, and images demonstrating demarcated ROI as per the described method.

Anatomical boundaries for ROIs can be difficult to define and are a point for discussion. Specifically, whether ROIs should include what might be considered extra-muscular fat. To rationalise our approach, we consider that if fat is occupying space within the epimysia of either ES or SPT, it is potentially compromising the functional integrity of that muscle tissue and should be included in the ROI. For example, fat that is occupying space that approximates bony tissue (spinous processes, laminae, zygapophyseal joint, and transverse processes) where the epimysium abuts, is included in the ROI. We base this decision in part on the findings for the lumbar spine of He et al. [46] who have shown this definition to have superior clinical relevance to the alternative non-inclusive definition. In addition, our wider group has recently shown improved repeatability for defining MF over ES, which we speculate is related to ease of definition based on MF approximating these bony landmarks [34]. Further, research has shown improved inter- and intra-rater reliability when following the spinous process and/or lamina in the cervical spine, while retaining the ability to discriminate between clinical groups [47].

Including fat that approximates the muscle at non-bony borders, particularly posteriorly where the definition between the thoracolumbar fascia can be variable, we err on the side of non-inclusivity. Our overarching rationale for this is that we intend to capture tissues that are or were, muscular, and not tissues that were unlikely to have a muscular origin. There is clear distinction of the rounded epimysium that encapsulates iliocostalis, longissimus, and the SPT, with small fascial ‘tents’ in the posterior border between each; these ‘tents’ are typically fat-filled (see Fig. 1d insert). The vast majority of investigators have employed this ROI definition (see Table 1), which allows for comparisons between studies. In using a different approach, Shahidi et al. [48] recently describe their posterior border for MF and ES as following the margin of the thoracolumbar fascia. However, their definition and representative figure indicate that this ROI includes a significant volume of fat extraneous (posterior and lateral) to the epimysium of the lumbar ES and SPT muscles. We contend that this space does not contain any muscle tissue, and therefore should not be included in measurement that seeks to provide comment on the functional capacity or quality of contractile elements such as the spinal muscles. We appreciate there may be strong value in recording the quantity and spatial distribution of this fatty deposition in terms of clinical relevance and potential to impede adjacent muscle function. However, we would suggest this fat be captured as a separate ROI rather than considered with any measure of muscle quality involving the paravertebral muscles.

Rater experience of anatomy, particularly cadaveric or three-dimensional, could influence the definition of lumbar paravertebral musculature on axial MRI images (e.g. greater experience results in less error). However, larger numbers of raters with varying levels of anatomical and post-production MRI experience would be required before deriving definitive conclusions on the influence of experience in quantifying MFI. Two studies examining the influence of rater experience on quantifying MFI in lumbar paravertebral muscles have indicated comparably high repeatability between novice and experienced raters given adequate training and practise of the novice rater [34, 45].

Challenges for the consistent and accurate quantification of MFI exist. These include, but are not limited to, a wide-variety of available whole-body human MRI scanners worldwide and the potential for varying results across field strengths (e.g. 1.5 Tesla to 3 Tesla). Accordingly, the question of how best to optimise the analysis of lumbar paravertebral muscle composition is a consideration that warrants comment. It is reasonable to assume that the higher the magnetic field (e.g. 3 Tesla versus 1.5 Tesla) the better the signal-to-noise and contrast-to-noise, but this is overly simplistic. While the quantification of muscle fat with MRI (and the Dixon sequence) is not without some complexities, we feel such discussion is beyond the scope of this paper. However, based on the available literature [49], discussions with experts in the field, experiential, and empirical evidence [26, 50], the authors opine that 1.5 Tesla and above is suitable when using the proposed MFI quantification method.

While methods employing a single MR slice are time efficient in determining fat proportion within an ROI, typically based on cross sectional area (mm^2^), the MR slice has a thickness [14, 15]. Accordingly, volumetric measures are more appropriate and have shown to be more meaningful functionally [47, 51]. We therefore recommend a multi-slice approach that derives fat content based on a three-dimensional volume across L1-L5 (or the levels of interest). It is acknowledged that acquiring such data is time-consuming, even using semi-automated or automated programmes. By way of potential compromise toward a time-efficient capture of lumbar paravertebral MFI on the basis of volume, Crawford et al. [5] have shown that the fat content at L4 best represents that of the entire lumbar region in healthy participants. Measuring multiple slices at this level alone may present an effective option in busy clinical radiology environments, although further data are required to support the validity and reliability of the technique. Research efforts should strive to comprehensively include the entire lumbar spine toward a stronger body of evidence regarding age-aggregated lumbar paravertebral muscle composition, which should be undertaken for healthy volunteers, and in asymptomatic and symptomatic disease cohorts.

## Conclusion

We present a foundational method for defining the ROIs of the lumbar paravertebral muscles MF and ES from axial MRIs. This includes a detailed and step-by-step description of the proposed technique to facilitate accurate reproduction of the method. This method has been proposed to provide a platform for standardizing measurement of lumbar paravertebral muscle ROI, with the aim of allowing accurate and reliable comparison of muscle quality between studies in (and beyond) this field.

## Abbreviations

ES: Erector Spinae
FCSA: Functional Cross Sectional Area
FI: Fatty infiltration
FSPGR: Fast-spoiled gradient echo
IP: In-phase (water)
L1,4: Lumbar levels L1 and L4
LBP: Low back pain
MF: Multifidus
MRI: Magnetic resonance imaging
OP: Opposed-phase (Fat)
PS: Psoas major
QL: Quadratus lumborum
RA: Rectus abdominis
ROI: Region of interest
SI: Signal intensity
SPT: Spinotransverse muscles
